# Prognostic significance of the C-reactive protein-to-lymphocyte ratio in patients with cancers

**DOI:** 10.3389/fonc.2026.1932997

**Published:** 2026-08-21

**Authors:** Xiaohui Liu, Shaobo Zhang, Yi Liu

**Affiliations:** 1 Department of Burns and Plastic Surgery & Wound Repair Surgery, the Second Hospital & Clinical Medical School, Lanzhou University, Lanzhou, China; 2 Department of Thoracic Surgery, the Second Hospital and Clinical Medical School, Lanzhou University, Lanzhou, China

**Keywords:** cancers, C-reactive protein-to-lymphocyte ratio, meta-analysis, prognosis, survival outcome

## Abstract

**Background:**

The C-reactive protein-to-lymphocyte ratio (CLR), a novel inflammation-based biomarker, has been reported to be associated with prognosis in various cancers. However, the prognostic significance of CLR remains controversial. Therefore, we performed a meta-analysis to comprehensively evaluate the prognostic value of CLR in patients with cancers.

**Methods:**

Relevant studies were systematically searched in PubMed, Embase, and Web of Science up to March 21, 2026. Pooled hazard ratios (HRs) with corresponding 95% confidence intervals (CIs) were calculated to assess the association between CLR and survival outcomes, including overall survival (OS), disease-free survival (DFS), and progression-free survival (PFS).

**Results:**

A total of 22 studies from 18 articles involving 7220 patients were included. The pooled analysis demonstrated that elevated CLR was significantly associated with poorer OS (HR:1.65, 95% CI: 1.43-1.91), PFS (HR:1.53, 95% CI: 1.15-2.05), and DFS (HR:1.67, 95% CI: 1.41-1.97). Subgroup analysis further confirmed the prognostic value of CLR across country, treatment method, sample size, cancer type and analysis type.

**Conclusions:**

Elevated CLR was significantly associated with unfavorable survival outcomes in cancers and may serve as a promising prognostic biomarker in clinical practice.

## Introduction

Cancer remains a major global health burden and a leading cause of morbidity and mortality worldwide (1). Despite considerable progress in cancer diagnosis and treatment, the prognosis of many patients remains unsatisfactory because of tumor recurrence, metastasis, and resistance to therapy (2). Therefore, the identification of reliable and cost-effective prognostic biomarkers is of great clinical importance.

Since Virchow first proposed the association between inflammation and cancer in the nineteenth century, chronic inflammatory responses have been recognized as one of the hallmarks of cancer development (3). Tumor-associated inflammatory responses can promote malignant progression through multiple biological mechanisms, including stimulation of angiogenesis, enhancement of tumor cell proliferation, inhibition of apoptosis, remodeling of the tumor microenvironment, and suppression of antitumor immunity (4, 5). Persistent systemic inflammation may also contribute to cancer cachexia, malnutrition, and immune dysfunction, ultimately leading to poor clinical outcomes in cancer patients (6, 7). In recent years, a variety of inflammation-based biomarkers have been investigated as prognostic indicators in malignancies (8–10). These markers are easily obtained from routine blood tests and reflect the balance between systemic inflammation and host immune status.

The C-reactive protein-to-lymphocyte ratio (CLR), a novel inflammation-based biomarker derived from serum C-reactive protein (CRP) levels and peripheral lymphocyte counts, has recently attracted increasing attention (11, 12). Recently, numerous studies have explored the prognostic significance of CLR in various cancers, including pancreatic cancer, colorectal cancer, gastric cancer, lung cancer, hepatocellular carcinoma, and nasopharyngeal carcinoma (13–18). Most studies have suggested that elevated CLR is associated with poorer survival outcomes, including reduced overall survival (OS), disease-free survival (DFS), and progression-free survival (PFS). Nevertheless, the reported findings remain inconsistent. Therefore, the present study was conducted to comprehensively investigate the association between CLR and survival outcomes in cancer patients through the meta-analysis.

## Methods

### Search strategy

A systematic literature search was conducted in the electronic databases including PubMed, Embase, and Web of Science up to March 21, 2026. The systematic search strategy used the following keywords: (“C-reactive protein-to-lymphocyte ratio” or “CRP-to-lymphocyte ratio” or “C-reactive protein/lymphocyte ratio” or “CRP/lymphocyte ratio” or “C-reactive protein to lymphocyte ratio” or “CRP to lymphocyte ratio”) AND (cancer or tumor or carcinoma or neoplasm or malignancy) AND (prognosis OR prognostic OR survival OR outcome). Language restrictions were set in English. The titles, abstracts, full texts, and the possible references were screened to identify qualified studies. In addition, the reference lists of all included articles and relevant reviews were manually screened to identify potentially eligible studies that may have been missed during the initial database search. The study was performed in accordance with the Preferred Reporting Items for Systematic Reviews and Meta-Analyses (PRISMA) guidelines. All procedures were independently completed by multiple investigators to minimize selection bias and improve the reliability of the pooled results.

### Inclusion and exclusion criteria

Studies were considered eligible if they met the following criteria: 1. Patients were pathologically or clinically diagnosed with cancers; 2.The study investigated the association between CLR and survival outcomes in cancer patients; 3.Hazard ratios (HRs) and corresponding 95% confidence intervals (CIs) could be directly obtained or indirectly calculated from the study; 4.Original studies with retrospective or prospective cohort designs were included. The exclusion criteria were as follows: 1.Reviews, case reports, conference abstracts, editorials, letters, comments, or animal studies;2.Duplicate publications or overlapping patient cohorts;3.Studies lacking sufficient survival data to estimate HRs and 95% CIs; 4.Non-English publications. When multiple studies involved overlapping patient populations, the most recent or most comprehensive study with the largest sample size was included.

### Data extraction and quality assessment

Data extraction was independently conducted by three investigators using a standardized data collection form. Disagreements occurring during the data screening and extraction process were resolved through team consensus. The following information was extracted from each eligible study: First author’s name, year of publication, country, tumor type, sample size, treatment methods, analysis types and survival outcomes. When both univariate and multivariate analyses were reported, the multivariate results were preferentially extracted because they accounted for potential confounding factors and provided more reliable prognostic estimates. The methodological quality of the included studies was evaluated using the Newcastle-Ottawa Scale (NOS) (19). The total NOS score ranges from 0 to 9 points. Studies with NOS scores of 7–9 were considered high quality, scores of 4–6 were regarded as moderate quality, and scores of 0–3 indicated low quality.

### Statistical analysis

All data analyses were performed using the STATA version 12.0 software (Stata Corporation, College Station, TX, USA). HRs and their corresponding 95% CIs were used to evaluate the association between elevated CLR and survival outcomes in cancer patients. Statistical heterogeneity among studies was evaluated using Cochran’s Q test and Higgins’ I² statistic. The I² value >50% or a P-value <0.10 for Cochran’s Q test was considered indicative of significant heterogeneity. In the presence of substantial heterogeneity, a random-effects model (DerSimonian–Laird method) was applied. Otherwise, a fixed-effects model (Mantel–Haenszel method) was used. Subgroup analysis and meta-regression were performed to further explore the heterogeneity. Sensitivity analysis was used to test the stability of the results.Publication bias was assessed using Begg’s test and Egger’s test (20). When publication bias was detected, the trim-and-fill method was further applied to evaluate the influence of publication bias on the pooled analysis (21). P <0.05 was considered statistically significant.

## Results

### Search results

**Figure 1:**
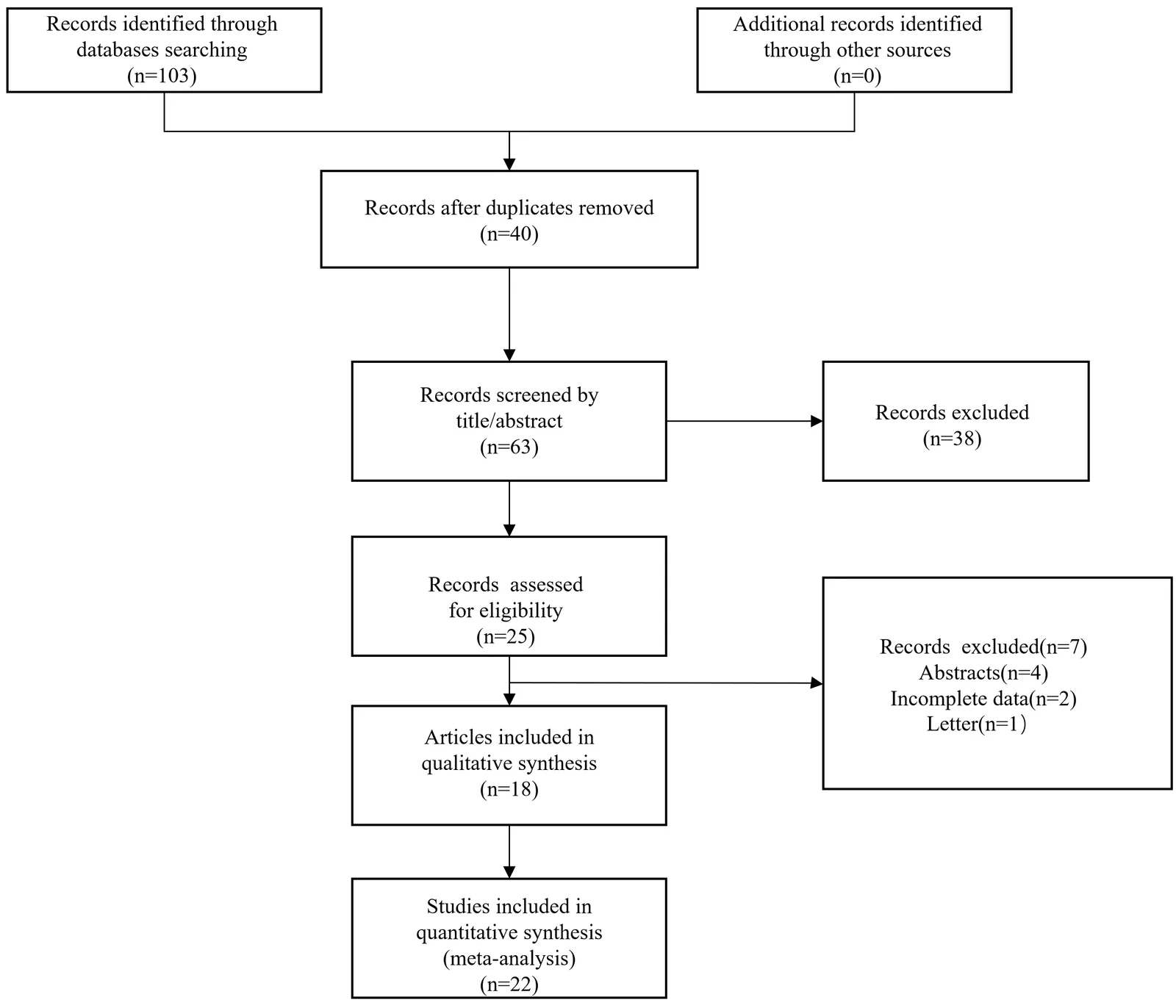
Flow diagram for study screening and selection processes.

Initially, a total of 103 potentially relevant articles were identified through electronic database searches. After removing 40 duplicate records, 63 studies remained for preliminary screening. Subsequently, titles and abstracts were carefully reviewed, resulting in the exclusion of 38 studies. The remaining 25 full-text articles were further assessed for eligibility. Among these, 7 studies were excluded. Finally, 18 articles published between 2020 and 2026 were included in the final meta-analysis (13, 14, 16, 18, 22–35). The detailed study selection process was illustrated in **Figure 1**.

### Study characteristics

The total number of patients in the included articles was 7220 (range: 23–2471 patients).12 studies were conducted in China, 5 studies were conducted in Japan, and 2 studies were conducted in Egypt. The remaining 3 studies were conducted in Turkey, Korea and Germany. 21 studies reported overall survival (OS) data, 5 studies displayed disease-free survival (DFS) data, 7 studies covered progression-free survival(PFS). A total of 6 different cancers were included, such as cervical cancer, lung cancer, pancreatic cancer, nasopharyngeal carcinoma, head and neck cancer,melanoma,oral cancer,esophageal cancer,gastric cancer, soft tissue sarcoma, colorectal cancer and neuroblastoma. Most studies used multivariate cox proportional hazards models to assess prognostic significance, thereby reducing the influence of confounding factors. According to the NOS, the quality scores of the included studies ranged from 6 to 8, with a mean score of 6.81, indicating that the methodological quality of most studies was moderate to high. The basic information was shown in **Table 1**.

**Table 1:** The basic information of the included studies.

Study	Country	Design	Cancer type	Treatment methods	Sample size	Analysis type	Survival analysis	NOS score
Buyukbayram 2024 (22)	Turkey	R	Cervical cancer	Fixed	90	MVA	OS,PFS	7
Chen 2025 (23)	China	R	Lung cancer	Radiotherapy	23	UVA	OS,PFS	6
Fan 2020 (24)	China	R	Pancreatic cancer	Fixed	997	MVA	OS	7
Han2020 (18)	China	R	Nasopharyngeal carcinoma	Radiotherapy	162	MVA	OS,PFS	7
Hwang 2021 (25)	Korea	R	Lung cancer	Surgery	331	MVA	OS	7
Jungbauer 2026 (26)	Germany	R	Head and neck cancer	Immunotherapy	49	UVA	OS	6
Kang 2025 (27)	China	R	Melanoma	Surgery	36	MVA	OS	7
Ko 2021 (28)	China	R	Oral cancer	Surgery	337	MVA	OS,DFS	8
Li 2023A (29)	China	R	Esophageal cancer	Chemotherapy	38	UVA	OS,PFS	6
Li 2023B (29)	China	R	Gastric cancer	Chemotherapy	54	UVA	OS,PFS	6
Matsui2022 (30)	Japan	R	Soft tissue sarcoma	Surgery	113	MVA	OS	7
Meng 2021 (31)	China	R	Colorectal cancer	Surgery	2471	MVA	OS	7
Nagano 2023A (32)	Japan	R	Lung cancer	Surgery	647	MVA	OS,DFS	8
Nagano 2023B (32)	Japan	R	Lung cancer	Surgery	647	MVA	OS,DFS	8
Nagano 2023B (32)	Japan	R	Lung cancer	Surgery	408	MVA	OS,DFS	8
Nooh 2022A (33)	Egypt	R	Cancers	Fixed	22	UVA	OS	6
Nooh 2022B (33)	Egypt	R	Cancers	Fixed	62	UVA	OS	6
Taniai 2020	Japan	R	Colorectal cancer	Surgery	197	MVA	OS,DFS	7
Wei 2025 (13)	China	R	Pancreatic cancer	Surgery	171	MVA	OS	6
Wu 2025 (34)	China	R	Neuroblastoma	Surgery	201	MVA	OS,PFS	6
Zhang 2026 (35)	China	R	Cancers	Fixed	47	MVA	OS	7
Sheng 2025 (16)	China	R	Lung cancer	Fixed	117	MVA	PFS	7

R, retrospective; OS, overall survival; DFS, disease-free survival; PFS, progression-free survival, MVA, multivariate analysis;UVA, univariate analysis; NOS score, Newcastle-Ottawa Scale score.

### Association between elevated CLR and OS

**Figure 2:**
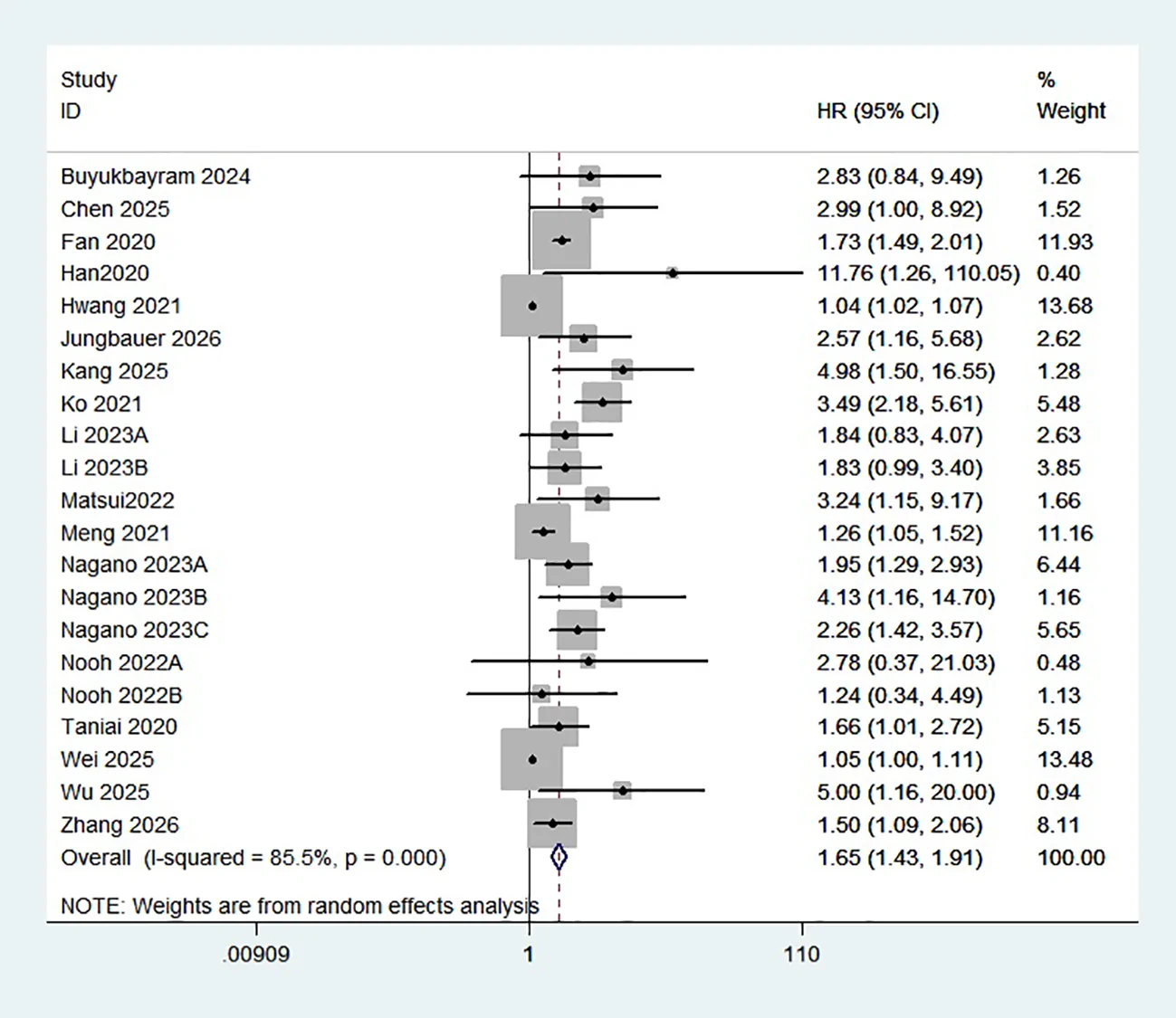
Forest plot of the association between high CLR and OS.

A total of 21 studies from 15 articles involving patients with various cancers evaluated the association between elevated CLR and OS. Because significant heterogeneity was observed among studies, a random-effects model was applied for pooled analysis (I^2^ = 85.5%). The pooled results revealed that elevated CLR was significantly associated with poorer OS in patients with cancers (HR:1.65,95% CI: 1.43-1.91). These findings indicated that patients with high CLR had a 65% increased risk of mortality compared with those with low CLR. The corresponding forest plot was presented in **Figure 2**.

### Subgroup analysis and meta-regression for OS

To further evaluate the prognostic stability of CLR and identify potential sources of heterogeneity, subgroup analysis and meta-regression were conducted according to cancer type, treatment methods, analysis type, sample size and country (**Table 2**). Subgroup analysis demonstrated that elevated CLR was consistently associated with poorer survival outcomes across most subgroups. Stratified by country, significant prognostic effects were observed in studies from China, Japan, Germany, and Korea, whereas studies from Egypt showed no statistically significant association. According to treatment modality, the prognostic impact remained significant in patients receiving surgery, radiotherapy, chemotherapy, and immunotherapy, with the strongest effect observed in the radiotherapy subgroup (HR:3.90, 95% CI: 1.46-10.39). Regarding sample size, both studies with fewer than 300 patients and those with more than 300 patients demonstrated significantly worse survival outcomes. In cancer-specific subgroup analyses, significant associations were identified in lung cancer, digestive system cancers, head and neck cancers, and other malignancies, with head and neck cancers showing a particularly strong prognostic effect (HR: 3.36, 95% CI: 2.25-5.01). Furthermore, both multivariate and univariate analyses confirmed the unfavorable prognostic significance of the biomarker. Subgroup analysis revealed that country, treatment method, sample size, cancer type, and analysis type were potential sources of heterogeneity. Meta-regression analysis indicated that cancer type might partially explain the observed heterogeneity (P = 0.042).

**Table 2:** Subgroup analysis and meta-regression for OS.

Factors	Studies	HR (95%)	P	Heterogeneity		Meta-regression		
				I^2^	P	tau2	Adj R (%)	P
Country						0.1468	-8.06	0.81
China	13		<0.01	86.7	<0.01			
Japan	4	2.23 (1.67-2.97)	<0.01	0	0.611			
Turkey	1	2.83 (0.84-9.49)						
Germany	1	2.57 (1.16-5.68)						
Egypt	2	1.56 (0.53-4.63)	0.42	0	0.51			
Korea	1	1.04 (1.02-1.07)						
Treatment method						0.1299	-1.59	0.454
Fixed	5	1.69 (1.48-1.94)	<0.01	0	0.773			
Surgery	11	1.46 (1.26-1.69)	<0.01	86.1	<0.01			
Radiotherapy	2	3.90 (1.46-10.39)	0.007	13.9	0.281			
Chemotherapy	2	1.84 (1.13-2.99)	0.014	0	0.992			
Immunotherapy	1	2.57 (1.16-5.68)						
Sample size						0.14	-10.74	0.67
<300	14	2.01 (1.46-2.77)	<0.01	69.8	<0.01			
>300	7	1.79 (1.31-2.44)	<0.01	93.7	<0.01			
Cancer type						0.11	13.95	0.042
Lung cancer	5	1.94 (1.14-3.29)	0.015	85.6	<0.01			
Digestive system cancer	6	1.44 (1.10-1.88)	0.007	89.2	<0.01			
Head and neck cancer	3	3.36 (2.25-5.01)	<0.01	0	0.434			
Cancers	3	1.50 (1.10-2.04)	0.01	0	0.802			
Others	4	3.77 (2.07-6.85)	<0.01	0	0.884			
Analysis type						0.1287	-0.6	0.66
MVA	15	1.97 (1.53-2.53)	<0.01	87.6	<0.01			
UVA	6	2.05 (1.42-2.95)	<0.01	0	0.896			

MVA, multivariate analysis; UVA, univariate analysis.

### Association between elevated CLR and DFS/PFS

**Figure 3:**
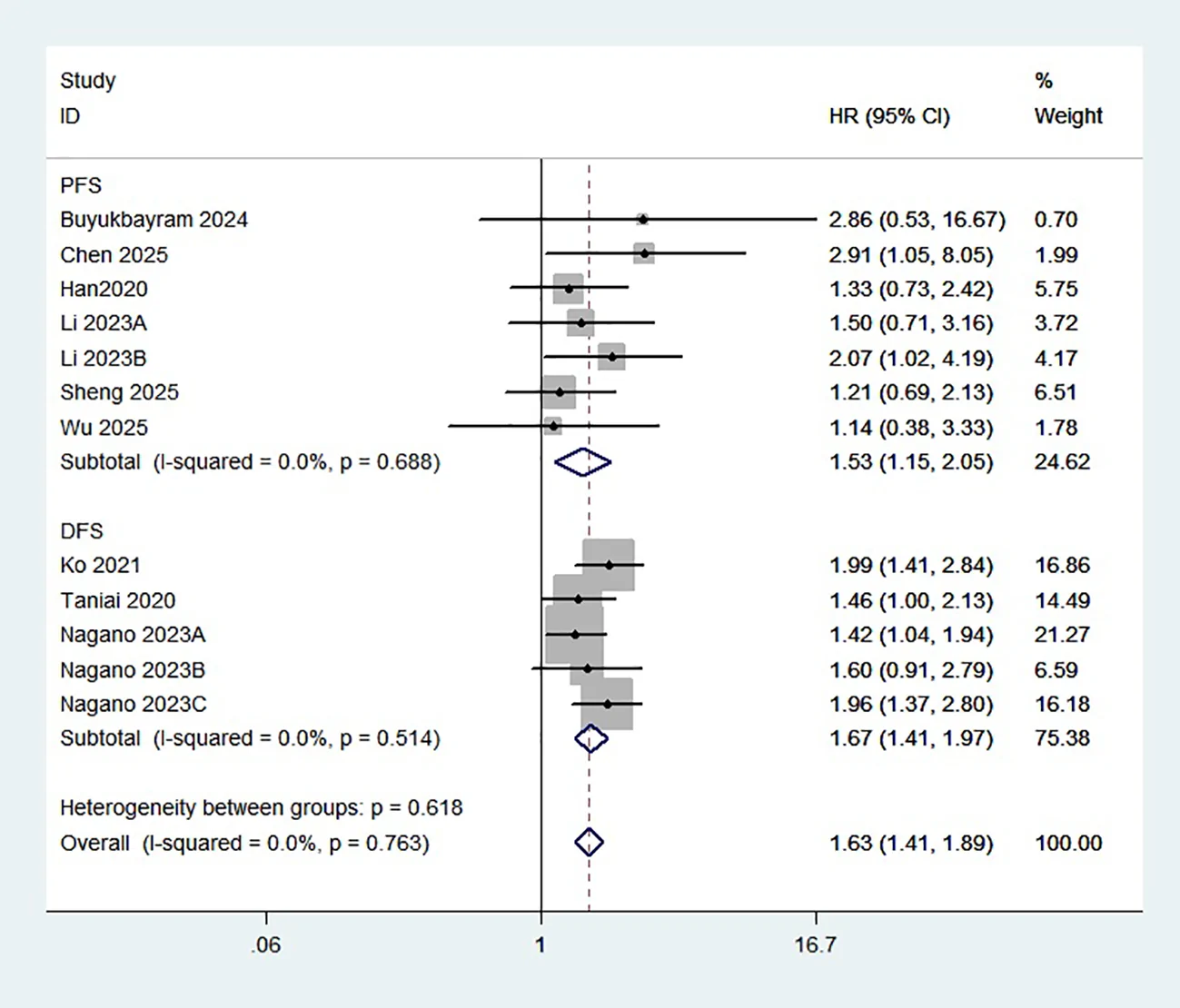
Forest plot of the association between high CLR and DFS/PFS.

A total of 12 studies from 9 articles investigated the association between CLR and DFS/PFS. Because no obvious heterogeneity was detected among these studies, a fixed-effects model was applied (I^2^ = 0%). The pooled analysis demonstrated that elevated CLR was significantly associated with unfavorable DFS/PFS (HR: 1.63; 95% CI: 1.41-1.89). Furthermore, the subgroup analysis revealed that patients with high CLR generally exhibited poorer PFS (HR:1.53,95% CI:1.15-2.05) and DFS (HR:1.67,95% CI:1.41-1.97). These results suggested that elevated CLR may predict not only increased mortality risk but also higher risks of recurrence and progression in patients with cancer. The forest plot was illustrated in **Figure 3**.

### Sensitivity analysis

**Figure 4:**
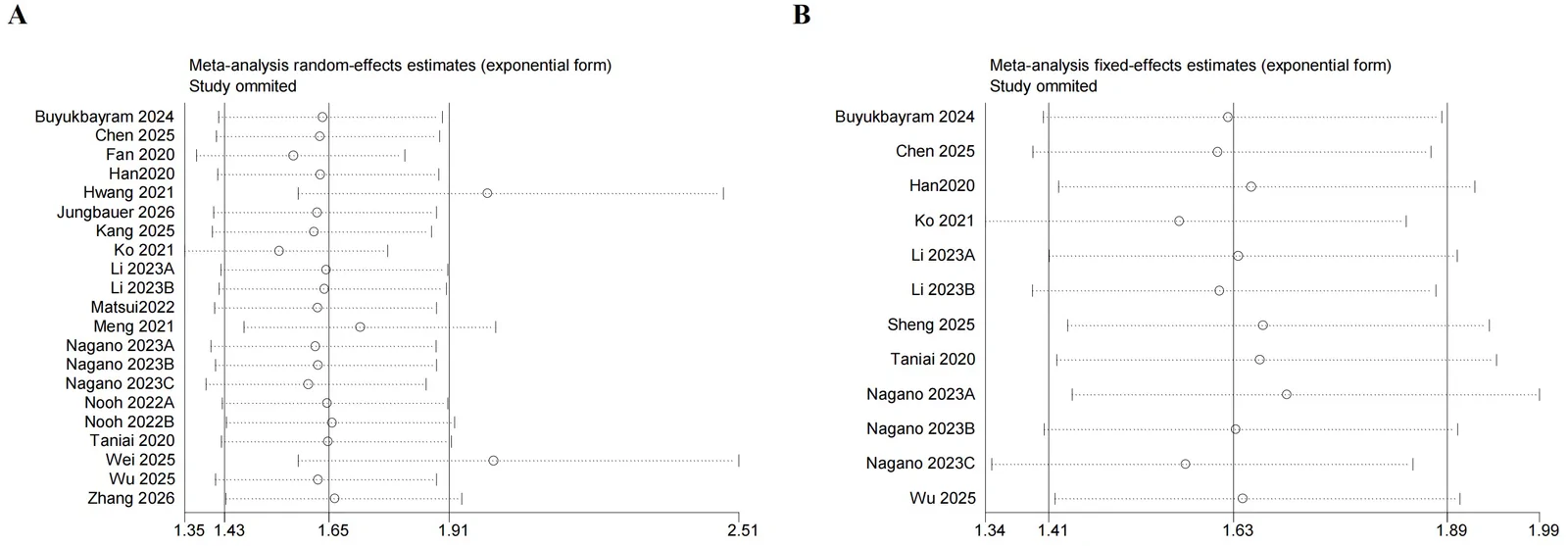
Leave-one-out sensitivity analysis for **(A)** OS and **(B)** DFS/PFS.

Sensitivity analysis was conducted using a leave-one-out approach to evaluate the stability and reliability of the pooled results for OS and DFS/PFS. Each study was sequentially excluded, and the pooled HRs were recalculated. The results demonstrated that omission of any single study did not substantially alter the overall pooled HRs, indicating that the prognostic significance of CLR was stable and robust. No individual study exerted a disproportionate influence on the pooled estimates. The sensitivity analysis plots are shown in **Figures 4A, B**.

### Publication bias

**Figure 5:**
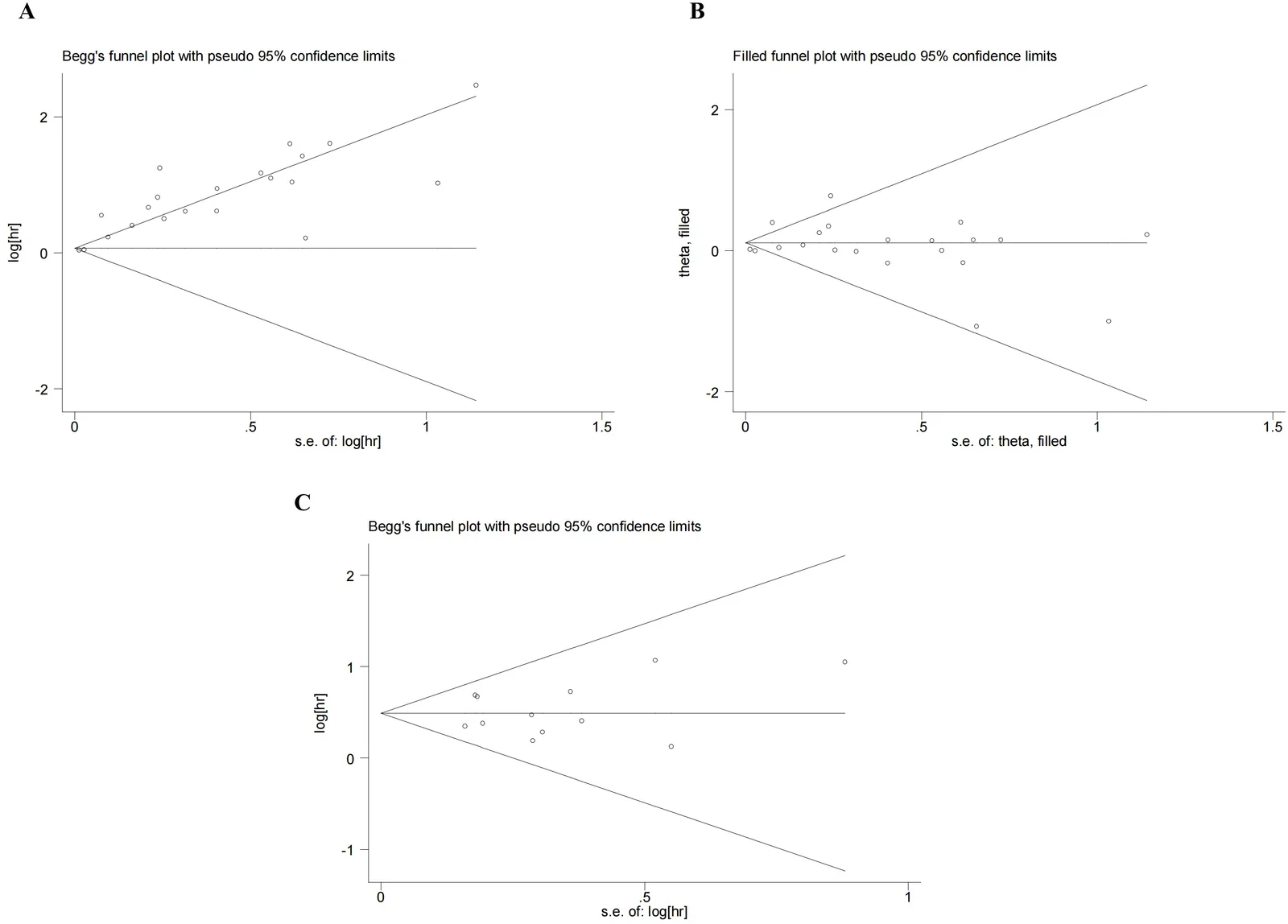
Publication bias. **(A)** publication bias for OS. **(B)** trim-and-fill method for OS. **(C)** publication bias for DFS/PFS.

Publication bias was assessed using Begg’s test and Egger’s test. For OS, the P value of Begg’s test showed no statistically significant publication bias (P = 0.833). However, Egger’s test indicated potential publication bias(P<0.01)(**Figure 5A**).Therefore, the trim-and-fill method was further applied to assess the influence of potentially missing studies. After adjustment, the pooled results remained statistically significant. These findings suggested that the prognostic significance of CLR for OS remained stable despite potential publication bias (HR:1.12,95%CI:1.03-1.22) (**Figure 5B**). P values of Begg’s and Egger’s tests for DFS/PFS were 0.945 and 0.756, respectively (**Figure 5C**). P was more than 0.05 and no significant bias was observed.

## Discussion

Systemic inflammation has increasingly been recognized as a key hallmark of cancer progression and an important determinant of clinical outcomes in oncology. In recent years, numerous inflammation-based biomarkers have been proposed as prognostic indicators in various malignancies. Among these, CLR integrated inflammatory and immune parameters, has gained increasing attention due to its simplicity, low cost, and accessibility in routine clinical practice. In this study, we performed a comprehensive meta-analysis of 22 studies involving 7,220 patients to evaluate the prognostic value of CLR in cancers. The pooled results showed that elevated CLR was significantly associated with poorer OS, DFS, and PFS, suggesting that CLR may serve as a potential prognostic biomarker in patients with malignancies. To our knowledge, the study was the first meta-analysis to systematically assess the prognostic role of CLR across multiple cancer types. Compared with individual retrospective studies, our findings provide more robust evidence by integrating data from diverse populations and tumor types. Importantly, subgroup analyses consistently demonstrated that elevated CLR was associated with unfavorable prognosis across different cancer type, treatment method, and country, supporting the robustness of the overall findings.

Although the pooled results consistently supported the prognostic value of CLR, significant heterogeneity was observed in the OS analysis. Several factors may account for the observed heterogeneity. Firstly, the included studies encompassed a wide range of tumor types with distinct biological characteristics, clinical behaviors, and prognostic patterns. Given that the degree of systemic inflammation and immune dysregulation varies substantially across different cancers, the prognostic impact of CLR may not be entirely uniform among cancer types. Secondly, considerable variation existed in the cutoff values used to define elevated CLR, which may have influenced patient stratification and contributed to differences in prognostic estimates. Thirdly, heterogeneity in patient characteristics, including age distribution, disease stage, performance status, comorbidities, and nutritional condition, may have further affected survival outcomes. In addition, differences in treatment strategies, such as surgery, chemotherapy, radiotherapy, targeted therapy, and immunotherapy, could influence both systemic inflammatory responses and clinical prognosis, thereby contributing to variability across studies. Notably, our meta-regression analysis suggested that cancer type was a potential source of heterogeneity, indicating that tumor-specific biological differences may partially explain the observed variability.

The biological mechanisms underlying the prognostic significance of CLR are likely multifactorial and closely related to the interaction between systemic inflammation, immune dysfunction, and tumor progression. CLR is composed of two important laboratory parameters: serum CRP and peripheral lymphocyte count. These two variables reflect opposite aspects of host-tumor interactions, including inflammatory activation and antitumor immune capacity. We attempted to analyze the potential mechanism underlying its prognostic value by examining the composition of the CLR.

CRP is a classical acute-phase protein synthesized by hepatocytes under the stimulation of inflammatory cytokines (36). Elevated CRP levels are generally considered indicators of systemic inflammatory activation (37). Inflammatory cytokines can promote tumor cell proliferation, angiogenesis, epithelial-mesenchymal transition, invasion, and metastasis (38). Furthermore, persistent inflammatory responses may remodel the tumor microenvironment and facilitate immune escape (39). Previous studies have shown that elevated CRP levels are significantly associated with advanced tumor stage, cachexia, malnutrition, and poor survival outcomes in various cancers (40–42). In addition to reflecting systemic inflammation, CRP may also directly influence tumor biology. CRP can enhance tumor proliferation and metastasis by activating inflammatory signaling pathways and promoting angiogenesis (43). Elevated CRP levels have also been associated with reduced infiltration of CD4+ and CD8+ T lymphocytes within the tumor microenvironment, thereby weakening antitumor immune responses (44–46). Therefore, increased CRP levels may reflect both enhanced tumor-associated inflammation and impaired immune surveillance.

Lymphocytes are essential components of the adaptive immune system and play a central role in antitumor immunity. Tumor-infiltrating and circulating lymphocytes, particularly CD4+ helper T cells, CD8+ cytotoxic T cells, and natural killer cells, contribute to the recognition and elimination of malignant cells (47). Adequate lymphocyte levels are important for maintaining effective immune surveillance and suppressing tumor progression (48). In contrast, lymphocytopenia may indicate impaired host immune function and weakened antitumor immune responses, thereby facilitating tumor growth, invasion, and metastasis (49, 50). Previous studies have demonstrated that decreased lymphocyte counts are significantly associated with advanced tumor stage, recurrence, treatment resistance, and poor survival outcomes in different cancers (51, 52).

By integrating CRP and lymphocyte counts, CLR may simultaneously reflect systemic inflammatory burden and host immune status more comprehensively than either parameter alone. Elevated CLR, characterized by increased CRP levels and decreased lymphocyte counts, may indicate enhanced inflammatory response, impaired antitumor immunity, poor nutritional status, and aggressive tumor biology. Consequently, patients with elevated CLR are more likely to experience tumor progression, recurrence, metastasis, and unfavorable survival outcomes.

Several limitations of the present study should be acknowledged. Firstly, most included studies were retrospective analyses, which may introduce inherent selection bias and limit causal inference. Secondly, the sample sizes of several studies were relatively small, potentially reducing statistical power. Thirdly, some HRs and corresponding 95% CIs were indirectly extracted from Kaplan-Meier survival curves rather than directly reported, which may introduce estimation bias. Fourthly, although CLR demonstrated consistent prognostic significance across multiple cancer types, substantial clinical heterogeneity should be acknowledged. The included malignancies differed considerably in biological behavior, disease progression patterns, treatment strategies, and baseline prognosis, which may limit the generalizability of the pooled findings. Furthermore, survival outcomes in cancer patients are influenced by numerous factors beyond systemic inflammation, including tumor stage, performance status, nutritional status, comorbidities, molecular characteristics, and treatment-related variables. Fifthly, most included studies were conducted in Asian populations, particularly China and Japan. Differences in genetic background, lifestyle factors, healthcare systems, and baseline immune profiles among ethnic groups may influence peripheral lymphocyte levels and systemic inflammatory responses. Therefore, multicenter studies involving diverse geographic and ethnic populations were warranted to further validate the prognostic value and clinical applicability of CLR. Sixthly, lymphocyte counts may be affected by anticancer treatments, including chemotherapy, radiotherapy, targeted therapy, and immunotherapy. Treatment-induced lymphopenia may alter CLR values independently of tumor-related inflammatory responses, potentially introducing additional variability among studies. Future investigations should consider treatment timing and longitudinal changes in lymphocyte counts to better clarify the clinical significance of CLR. Seventh, differences in CRP detection methods may affect CRP values and subsequently influence CLR calculation and comparability across studies. Future research should establish standardized CRP measurement protocols and reporting criteria to improve the reproducibility and clinical applicability of CLR. In addition, it should be noted that the current evidence primarily supported CLR as a prognostic biomarker associated with survival outcomes rather than an independent predictive biomarker for treatment response. Although changes in inflammatory and immune parameters during treatment may potentially reflect therapeutic effects, whether CLR can predict treatment sensitivity or guide treatment selection remains uncertain and requires validation in prospective studies. Finally, another important challenge was the absence of a universally accepted CLR cutoff value. Different studies applied various approaches for determining CLR thresholds, resulting in limited comparability between studies. Future prospective multicenter studies should aim to establish standardized and clinically applicable CLR thresholds.

Despite these limitations, this study also possessed several important strengths. Firstly, it was the first and most comprehensive meta-analysis evaluating the prognostic significance of CLR across different cancers. Secondly, the pooled sample size was relatively large, improving the statistical reliability of the findings. Thirdly, comprehensive subgroup analyses, sensitivity analyses, and publication bias assessments were performed to enhance the robustness of the conclusions. Finally, CLR was a simple, inexpensive, and readily available laboratory biomarker that may have considerable clinical applicability in routine practice.

In conclusion, the present meta-analysis demonstrated that elevated CLR was significantly associated with unfavorable survival outcomes in cancers. As an easily obtainable blood-based biomarker, CLR could help clinicians identify high-risk patients before treatment initiation and facilitate risk stratification, treatment selection, and follow-up planning. Patients with elevated CLR may require closer surveillance, more aggressive therapeutic strategies, or intensified supportive care. Furthermore, dynamic monitoring of CLR during treatment may potentially assist in evaluating treatment response and disease progression. However, before CLR can be widely adopted in clinical practice, prospective large-scale multicenter studies were still needed to establish standardized cut-off values of CLR and validate its prognostic utility across different and treatment settings.

## Data Availability

The original contributions presented in the study are included in the article/supplementary material. Further inquiries can be directed to the corresponding author.

## References

[1] Sung H Ferlay J Siegel RL Laversanne M Soerjomataram I Jemal A . Global cancer statistics 2020: GLOBOCAN estimates of incidence and mortality worldwide for 36 cancers in 185 countries. CA Cancer J Clin. (2021) 71:209–49. doi: 10.3322/caac.21660 33538338

[2] Bray F Laversanne M Sung H Ferlay J Siegel RL Soerjomataram I . Global cancer statistics 2022: GLOBOCAN estimates of incidence and mortality worldwide for 36 cancers in 185 countries. CA Cancer J Clin. (2024) 74:229–63. doi: 10.3322/caac.21834 38572751

[3] Balkwill F Mantovani A . Inflammation and cancer: back to Virchow? Lancet. (2001) 357:539–45. doi: 10.1016/s0140-6736(00)04046-0 11229684

[4] Bi Q Wu JY Qiu XM Zhang JD Sun ZJ Wang W . Tumor-associated inflammation: the tumor-promoting immunity in the early stages of tumorigenesis. J Immunol Res. (2022) 2022:3128933. doi: 10.1155/2022/3128933 35733919 PMC9208911

[5] Coussens LM Werb Z . Inflammation and cancer. Nature. (2002) 420:860–7. doi: 10.1038/nature01322 12490959 PMC2803035

[6] Kiely M Lord B Ambs S . Immune response and inflammation in cancer health disparities. Trends Cancer. (2022) 8:316–27. doi: 10.1016/j.trecan.2021.11.010 34965905 PMC8930618

[7] Diakos CI Charles KA McMillan DC Clarke SJ . Cancer-related inflammation and treatment effectiveness. Lancet Oncol. (2014) 15:e493–503. doi: 10.1016/s1470-2045(14)70263-3 25281468

[8] Cupp MA Cariolou M Tzoulaki I Aune D Evangelou E Berlanga-Taylor AJ . Neutrophil to lymphocyte ratio and cancer prognosis: an umbrella review of systematic reviews and meta-analyses of observational studies. BMC Med. (2020) 18:360. doi: 10.1186/s12916-020-01817-1 33213430 PMC7678319

[9] Niu X Zhu Z Bao J . Prognostic significance of pretreatment controlling nutritional status score in urological cancers: a systematic review and meta-analysis. Cancer Cell Int. (2021) 21:126. doi: 10.1186/s12935-021-01813-2 33608012 PMC7893866

[10] Liu R Wang L Ye J Li X Ma W Xu X . Preoperative glasgow prognostic score was an effective prognostic indicator in patients with biliary tract cancer. Front Immunol. (2025) 16:1560944. doi: 10.3389/fimmu.2025.1560944 40264786 PMC12011749

[11] Sun X Lin J Wang Z Zhang C Zhao K Zhang X . Association between C-reactive protein to lymphocyte ratio and gallstones: a cross-sectional study. BMC Gastroenterol. (2025) 25:415. doi: 10.1186/s12876-025-04000-z 40442600 PMC12123812

[12] Ao T Huang Y Zhen P Hu M . Association between C-reactive protein to lymphocyte ratio and chronic obstructive pulmonary disease: a cross-sectional study. Int J Chron Obstruct Pulmon Dis. (2025) 20:1915–25. doi: 10.2147/copd.S510755 40529225 PMC12170868

[13] Wei Q Li Z Feng H Zhang J Wei L . Prognostic value of composite inflammatory prognostic model in pancreatic cancer. Mol Cell Probes. (2025) 84:102056. doi: 10.1016/j.mcp.2025.102056 41260275

[14] Taniai T Haruki K Hamura R Fujiwara Y Furukawa K Gocho T . The prognostic significance of C-reactive protein-to-lymphocyte ratio in colorectal liver metastases. J Surg Res. (2021) 258:414–21. doi: 10.1016/j.jss.2020.08.059 33109402

[15] Xu R Xiao S Ding Z Zhao P . The value of the C-reactive protein-to-lymphocyte ratio for predicting lymphovascular invasion based on nutritional status in gastric cancer. Technol Cancer Res Treat. (2022) 21:15330338221106517. doi: 10.1177/15330338221106517 35695253 PMC9201353

[16] Sheng S Wu Z Zheng H Zhang H Zhang Q Liu Z . Prognostic value of C-reactive protein-to-lymphocyte ratio in combined immunotherapy and chemotherapy for small cell lung cancer. J Inflammation Res. (2025) 18:9343–53. doi: 10.2147/jir.S517816 40692548 PMC12278946

[17] Liao M Chen P Liao Y Li J Yao W Sun T . Preoperative high-sensitivity C-reactive protein to lymphocyte ratio index plays a vital role in the prognosis of hepatocellular carcinoma after surgical resection. Onco Targets Ther. (2018) 11:5591–600. doi: 10.2147/ott.S167857 30237725 PMC6135434

[18] Han YY Chen KH Guan Y Chen L Lin MR Nong SK . Predictive value of some inflammatory indexes in the survival and toxicity of nasopharyngeal carcinoma. Cancer Manag Res. (2020) 12:11541–51. doi: 10.2147/cmar.S263100 33204165 PMC7667699

[19] Stang A . Critical evaluation of the Newcastle-Ottawa scale for the assessment of the quality of nonrandomized studies in meta-analyses. Eur J Epidemiol. (2010) 25:603–5. doi: 10.1007/s10654-010-9491-z 20652370

[20] Jung H Kang J Han KM Kim H . Prognostic value of Pentraxin3 protein expression in human Malignancies: a systematic review and meta-analysis. Cancers (Basel). (2024) 16:3754. doi: 10.3390/cancers16223754 39594709 PMC11593206

[21] Liu R Shen Y Cui J Ma W Wang J Chen C . Association between glucose to lymphocyte ratio and prognosis in patients with solid tumors. Front Immunol. (2024) 15:1454393. doi: 10.3389/fimmu.2024.1454393 39712026 PMC11662397

[22] Buyukbayram ME Hannarici Z Turhan A Caglar AA Esdur P Bilici M . A novel prognostic biomarker in progression free survival for patients with cervical cancer, glucose to C-reactive protein ratio (GCR). BMC Cancer. (2024) 24:626. doi: 10.1186/s12885-024-12347-x 38783223 PMC11112963

[23] Chen J Lin M Wang J Wang J Peng Y Yu Z . Prognostic significance of baseline systemic inflammation markers in PD-L1-negative advanced non-small cell lung cancer patients treated with the BRICS sequential regimen. Front Immunol. (2025) 16:1686521. doi: 10.3389/fimmu.2025.1686521 41451214 PMC12728061

[24] Fan Z Luo G Gong Y Xu H Qian Y Deng S . Prognostic value of the C-reactive protein/lymphocyte ratio in pancreatic cancer. Ann Surg Oncol. (2020) 27:4017–25. doi: 10.1245/s10434-020-08301-3 32144621

[25] Hwang JJ Hur JY Eo W An S Kim DH Lee S . Clinical significance of C-reactive protein to lymphocyte count ratio as a prognostic factor for survival in non-small cell lung cancer patients undergoing curative surgical resection. J Cancer. (2021) 12:4497–504. doi: 10.7150/jca.58094 34149913 PMC8210557

[26] Jungbauer F Ludwig S Huber L Affolter A Lammert A Rotter N . Systemic inflammation plays a key prognostic role in patients with head and neck cancer treated with immunotherapy and is linked to CT-based body composition metrics. Eur Arch Otorhinolaryngol. (2026) 283:3361–71. doi: 10.1007/s00405-026-10159-2 41838159 PMC13152974

[27] Kang D Su X Wang J Zhao L Huang R Zou Z . C-reactive protein to lymphocyte ratio (CLR) and lactate dehydrogenase to albumin ratio (LAR) as prognostic biomarkers in acral melanoma: association with tertiary lymphoid structures and immune cell infiltration. Cancer Med. (2025) 14:e71078. doi: 10.1002/cam4.71078 40716028 PMC12677929

[28] Ko CA Fang KH Hsu CM Lee YC Chang GH Huang EI . The preoperative C-reactive protein-lymphocyte ratio and the prognosis of oral cavity squamous cell carcinoma. Head Neck. (2021) 43:2740–54. doi: 10.1002/hed.26738 33991004

[29] Li N Gao L Ge Y Zhao L Bai C Wang Y . Prognostic and predictive significance of circulating biomarkers in patients with advanced upper gastrointestinal cancer undergoing systemic chemotherapy. Front Oncol. (2023) 13:1195848. doi: 10.3389/fonc.2023.1195848 37346066 PMC10280739

[30] Matsui Y Matsuda A Maejima A Shinoda Y Nakamura E Komiyama M . The clinical significance of perioperative inflammatory index as a prognostic factor for patients with retroperitoneal soft tissue sarcoma. Int J Clin Oncol. (2022) 27:1093–100. doi: 10.1007/s10147-022-02150-8 35319075

[31] Meng Y Long C Huang X Huang L Liao L Tang W . Prognostic role and clinical significance of C-reactive protein-lymphocyte ratio in colorectal cancer. Bioengineered. (2021) 12:5138–48. doi: 10.1080/21655979.2021.1960768 34436973 PMC8806856

[32] Nagano T Kinoshita F Hashinokuchi A Matsudo K Watanabe K Takamori S . Prognostic impact of C-reactive protein-to-lymphocyte ratio in non-small cell lung cancer: a propensity score-matching analysis. Ann Surg Oncol. (2023) 30:3781–8. doi: 10.1245/s10434-023-13250-8 36847957

[33] Nooh HA Abdellateif MS Refaat L Kandeel EZ Bayoumi A Samra M . The role of inflammatory indices in the outcome of COVID-19 cancer patients. Med Oncol. (2021) 39:6. doi: 10.1007/s12032-021-01605-8 34748094 PMC8573297

[34] Wu Q Xu C Cao Z Lv J Han Y Teng G . C-reactive protein-to-lymphocyte ratio and hemoglobin-to-red cell distribution width ratio as effective prognostic predictors in pediatric patients with neuroblastoma. Eur J Pediatr. (2025) 184:493. doi: 10.1007/s00431-025-06334-y 40689991

[35] Zhang Y Shan J Huang R Wei W Liu H . Improved survival with SBRT plus PD-1/PD-L1 inhibitors in lung metastases patients: a propensity score-matched study. Int Immunopharmacol. (2026) 174:116379. doi: 10.1016/j.intimp.2026.116379 41698294

[36] Pepys MB Hirschfield GM . C-reactive protein: a critical update. J Clin Invest. (2003) 111:1805–12. doi: 10.1172/jci18921 12813013 PMC161431

[37] Zhou HH Tang YL Xu TH Cheng B . C-reactive protein: structure, function, regulation, and role in clinical diseases. Front Immunol. (2024) 15:1425168. doi: 10.3389/fimmu.2024.1425168 38947332 PMC11211361

[38] Nishida A Andoh A . The role of inflammation in cancer: mechanisms of tumor initiation, progression, and metastasis. Cells. (2025) 14:488. doi: 10.3390/cells14070488 40214442 PMC11987742

[39] Greten FR Grivennikov SI . Inflammation and cancer: triggers, mechanisms, and consequences. Immunity. (2019) 51:27–41. doi: 10.1016/j.immuni.2019.06.025 31315034 PMC6831096

[40] Zhu M Ma Z Zhang X Hang D Yin R Feng J . C-reactive protein and cancer risk: a pan-cancer study of prospective cohort and Mendelian randomization analysis. BMC Med. (2022) 20:301. doi: 10.1186/s12916-022-02506-x 36117174 PMC9484145

[41] Woo HD Kim K Kim J . Association between preoperative C-reactive protein level and colorectal cancer survival: a meta-analysis. Cancer Causes Control. (2015) 26:1661–70. doi: 10.1007/s10552-015-0663-8 26376895

[42] Bruserud Ø Aarstad HH Tvedt THA . Combined C-reactive protein and novel inflammatory parameters as a predictor in cancer-what can we learn from the hematological experience? Cancers (Basel). (2020) 12:1966. doi: 10.3390/cancers12071966 32707721 PMC7409204

[43] Kim ES Kim SY Moon A . C-reactive protein signaling pathways in tumor progression. Biomol Ther (Seoul). (2023) 31:473–83. doi: 10.4062/biomolther.2023.132 37562952 PMC10468419

[44] Nakayama T Saito K Kumagai J Nakajima Y Kijima T Yoshida S . Higher serum C-reactive protein level represents the immunosuppressive tumor microenvironment in patients with clear cell renal cell carcinoma. Clin Genitourin Cancer. (2018) 16:e1151–8. doi: 10.1016/j.clgc.2018.07.027 30213543

[45] Hsu WL Hsieh YT Chen WM Chien MH Luo WJ Chang JH . High-fat diet induces C-reactive protein secretion, promoting lung adenocarcinoma via immune microenvironment modulation. Dis Model Mech. (2023) 16:dmm050360. doi: 10.1242/dmm.050360 37929799 PMC10651111

[46] Ravindranathan D Master VA Bilen MA . Inflammatory markers in cancer immunotherapy. Biol (Basel). (2021) 10:325. doi: 10.3390/biology10040325 33924623 PMC8069970

[47] Lei X Lei Y Li JK Du WX Li RG Yang J . Immune cells within the tumor microenvironment: biological functions and roles in cancer immunotherapy. Cancer Lett. (2020) 470:126–33. doi: 10.1016/j.canlet.2019.11.009 31730903

[48] Ikeda H Togashi Y . Aging, cancer, and antitumor immunity. Int J Clin Oncol. (2022) 27:316–22. doi: 10.1007/s10147-021-01913-z 33783658

[49] Ménétrier-Caux C Ray-Coquard I Blay JY Caux C . Lymphopenia in cancer patients and its effects on response to immunotherapy: an opportunity for combination with cytokines? J Immunother Cancer. (2019) 7:85. doi: 10.1186/s40425-019-0549-5 30922400 PMC6437964

[50] Koukourakis MI Giatromanolaki A . Lymphopenia and intratumoral lymphocytic balance in the era of cancer immuno-radiotherapy. Crit Rev Oncol Hematol. (2021) 159:103226. doi: 10.1016/j.critrevonc.2021.103226 33482348

[51] Gooden MJ de Bock GH Leffers N Daemen T Nijman HW . The prognostic influence of tumour-infiltrating lymphocytes in cancer: a systematic review with meta-analysis. Br J Cancer. (2011) 105:93–103. doi: 10.1038/bjc.2011.189 21629244 PMC3137407

[52] Liu Y Liu Z Yang Y Cui J Sun J Liu Y . The prognostic and biology of tumour-infiltrating lymphocytes in the immunotherapy of cancer. Br J Cancer. (2023) 129:1041–9. doi: 10.1038/s41416-023-02321-y 37452117 PMC10539364

